# A microbiome approach to sepsis: development and case-study application of novel methods for detection and isolation of microbes from whole blood

**DOI:** 10.1186/cc11798

**Published:** 2012-11-14

**Authors:** M Faria Crowder, JM Conly, MG Surette

**Affiliations:** 1 University of Calgary, Calgary, Canada; 2 University of Calgary, Snyder Institute for Chronic Diseases, Calgary, Canada; 3 McMaster University, Farncombe Family Digestive Health Research Institute, Hamilton, Canada

## Background

The application of molecular profiling methods to a wide variety of infections suggests that a polymicrobial community is much more common than suggested by standard clinical culture [1]. Our goal was to develop methods, using a microbiome approach, to improve culture and molecular diagnostics for bacterial sepsis.

## Methods

Culture and DNA extraction protocols were evaluated using synthetic bacterial communities inoculated into whole blood. Disruption of blood cells with a blood cell lysing detergent, with or without hypotonic osmotic shock, was carried out and evaluated for the ability to recover the community. Viable bacterial cells were recovered on solid media. Total DNA was examined by terminal-restriction fragment-length polymorphism (TRFLP) profiling. Efficiencies of recovery and limits of detection were determined. The optimized methodology was applied to clinical samples collected from consented patients in both the ICU and ED from two Calgary hospitals. Cultured organisms were identified by 16S rRNA gene sequencing. Molecular profiling was carried out using TRFLP and bacterial tag-encoded FLX amplicon pyrosequencing (bTEFAP).

## Results

Treatment of synthetic community organisms with a 5% wt/vol detergent added at a 1:1 or 1:5 ratio did not significantly impact their viability. TRFLP analysis indicated that the DNA from these communities could be recovered from whole blood following lysis and removal of host cells. Whole blood samples were analysed from septic patients. In three case studies we identified two to 16 bacterial species in the primary infection samples using direct culture and molecular methods. Conventional diagnostics only reported one organism. Molecular profiling of blood samples from these patients also identified correlating polymicrobial communities. Blood cultures for these samples were either negative (two of three cases) or monomicrobial (one of three cases), thereby underestimating the diversity seen with the TRFLP and bTEFAP analysis. These methods have been applied to 88 adult ICU blood samples, 20 primary infection samples, 36 adult ED samples, and seven pediatric ED samples with analysis ongoing.

## Conclusion

We have successfully developed a novel method to analyse whole blood in order to characterize the microbiome of sepsis infections. Preliminary results indicate that sepsis infections are polymicrobial in nature.
